# Muscarinic Receptors in Amygdala Control Trace Fear Conditioning

**DOI:** 10.1371/journal.pone.0045720

**Published:** 2012-09-21

**Authors:** Amber N. Baysinger, Brianne A. Kent, Thomas H. Brown

**Affiliations:** 1 Department of Psychology, Yale University, New Haven, Connecticut, United States of America; 2 Department of Cellular and Molecular Physiology, Yale School of Medicine, New Haven, Connecticut, United States of America; Pontifical Catholic University of Rio Grande, Brazil

## Abstract

Intelligent behavior requires transient memory, which entails the ability to retain information over short time periods. A newly-emerging hypothesis posits that endogenous persistent firing (EPF) is the neurophysiological foundation for aspects or types of transient memory. EPF is enabled by the activation of muscarinic acetylcholine receptors (mAChRs) and is triggered by suprathreshold stimulation. EPF occurs in several brain regions, including the lateral amygdala (LA). The present study examined the role of amygdalar mAChRs in trace fear conditioning, a paradigm that requires transient memory. If mAChR-dependent EPF selectively supports transient memory, then blocking amygdalar mAChRs should impair trace conditioning, while sparing delay and context conditioning, which presumably do not rely upon transient memory. To test the EPF hypothesis, LA was bilaterally infused, prior to trace or delay conditioning, with either a mAChR antagonist (scopolamine) or saline. Computerized video analysis quantified the amount of freezing elicited by the cue and by the training context. Scopolamine infusion profoundly reduced freezing in the trace conditioning group but had no significant effect on delay or context conditioning. This pattern of results was uniquely anticipated by the EPF hypothesis. The present findings are discussed in terms of a systems-level theory of how EPF in LA and several other brain regions might help support trace fear conditioning.

## Introduction

Intelligent behavior requires transient memory, loosely defined as the ability to retain information over short time periods. One successful paradigm for studying transient memory is trace conditioning [1]–[3]. In this Pavlovian procedure, a temporal gap, known as the trace interval, separates the offset of the conditional stimulus (CS) from the onset of the unconditional stimulus (US). If Pavlovian conditioning is supported by a contiguity-based form of synaptic plasticity [4]–[6], as is commonly assumed [7], [8], it follows that the nervous system must somehow maintain a transient representation of the CS until the occurrence of the US. The traditional hypothesis for transient memory proposes that information is temporarily maintained through reverberating activity in recurrent neural circuits [9], as originally proposed by Donald Hebb [10].

A newly-emerging hypothesis for an alternative or supplementary mechanism proposes that certain types or aspects of transient memory are supported by endogenous persistent firing (EPF) [11]–[25]. Persistent-firing neurons can continue to discharge for seconds to minutes after the termination of the initial depolarizing stimulus. By definition, EPF can be generated endogenously. Operationally, this means that it can occur during the blockade of ionotropic receptors for glutamate and GABA [11], [14], [17], [18], [20], [22], [23], [26], [27]. In several brain regions, EPF is enabled by activation of muscarinic cholinergic receptors (mAChRs) and can be elicited by a depolarizing current of sufficient strength and duration [11], [17], [18], [20]–[23], [26], [27]. The sustained inward current responsible for mAChR-dependent EPF is produced by a non-selective cation conductance whose gating depends on both elevated [Ca^2+^]_i_ and agonist binding to extracellular mAChRs [11], [14], [17], [18], [22], [23], [28], [29]. The ion pores responsible for this sustained conductance are thought to be comprised of subunits of TRPC channels [18], [22], [23], [28].

EPF has been well-established in brain slices of entorhinal cortex (EC) [24], the lateral nucleus of the amygdala (LA) [18], and perirhinal cortex (PR) [17]––three structures that are known to be essential for trace fear conditioning [15], [16], [30]–[33]. Most recently, persistent firing was also detected in the hippocampus (HC) [19], [26], [27], which has long been recognized as important for normal trace conditioning [32], [34], [35]. In support of the persistent-firing hypothesis, trace but not delay, fear conditioning is profoundly impaired by infusions of mAChR antagonists into PR [30], EC [16], and HC [36]. To evaluate the EPF hypothesis further, the present study examined whether mAChR activity in LA is also required for trace conditioning.

## Methods

### Subjects

Ninety male Sprague Dawley rats (260–290 g; Charles River Laboratories) were individually housed on a 12-h light cycle with *ad libitum* access to food and water. Rats were handled for 3–5 d prior to conditioning. All procedures were performed during the light cycle. Procedures were in strict compliance with the recommendations in the Guide for the Care and Use of Laboratory Animals of the National Institutes of Health. The protocol was approved by the Yale University Institutional Animal Care and Use Committee (License Number: 16-R-0001). Extra care was taken to minimize suffering of the animals.

### Surgery

Animals were anesthetized with ketamine (100 mg/kg) and xylazine (9 mg/kg) prior to surgery and secured in a stereotaxic apparatus (Stoelting). Guide cannulae (26 gauge; Plastics One) were lowered bilaterally into LA at the coordinates −3.0 posterior, ±5.3 lateral, and −7.0 ventral relative to bregma [37]. Cannulae were secured using dental cement (A-M Systems) and capped with stylets to prevent clogging. Animals recovered 5–7 d before conditioning.

### Cannula Infusion

LA was infused bilaterally (0.5 µl/side), with either sterile saline or scopolamine hydrobromide (10 mg/ml; Sigma-Aldrich), 15 to 20 min before conditioning. Infusers were constructed by connecting polyethylene tubing (0.38 mm inside diameter, A-M Systems) to an internal cannula (33 gauge, Plastics One), which protruded 1 mm past the tip of the guide cannula. The tubing was connected to a 10-µl Hamilton syringe. Infusions (0.1 µl/m/side) were delivered via a syringe pump (PHD 2000 Infusion; Harvard Apparatus). There was a 1-min rest period before removing the infusers.

### Conditioning Stimuli and Paradigms

**Figure 1:**
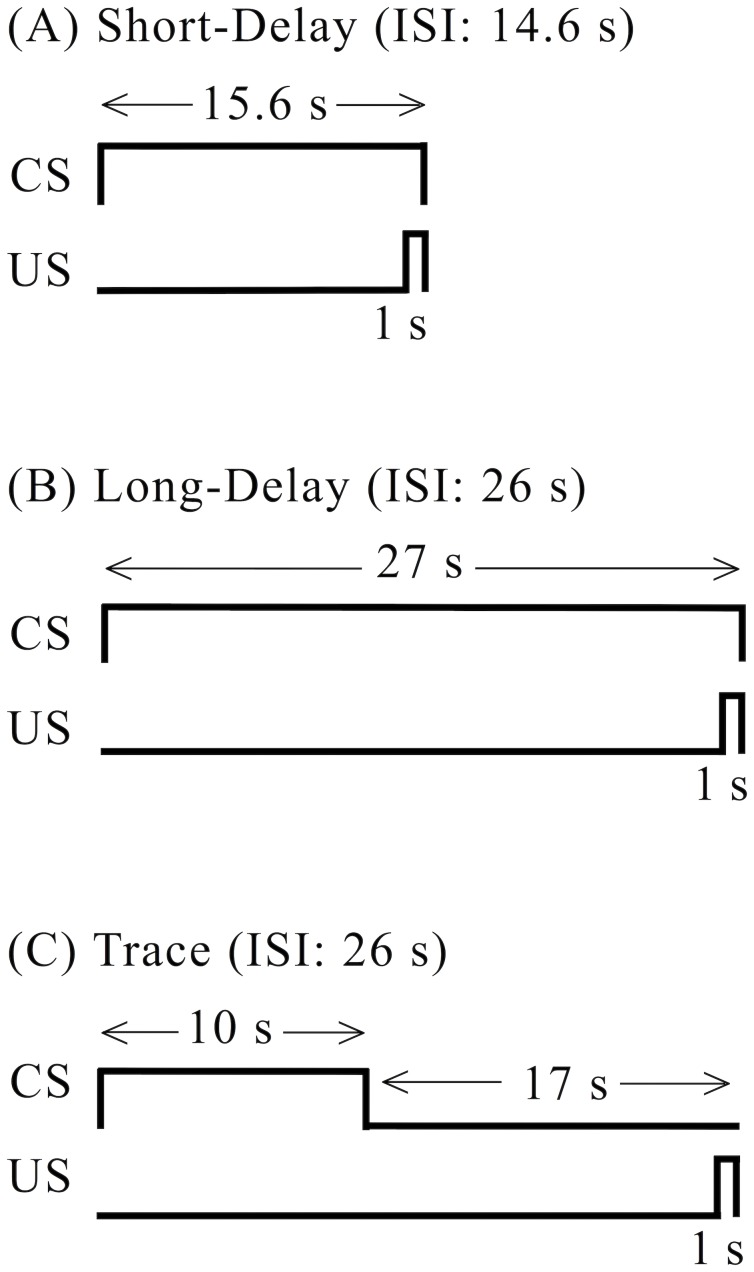
Three fear conditioning paradigms. (A) Short-delay conditioning. (B) Long-delay conditioning. (C) Trace conditioning. Note that the interstimulus interval (ISI) is the same in the long-delay and the trace paradigms. The CS-US relationships are matched to those used in a related study [30]. The CS was a 70-dB, 22-kHz tone and the US was a 1-s, 0.8-mA footshock.

Animals were randomly assigned to one of three conditioning paradigms (*n = *30 per paradigm), which are illustrated in Figure 1. Half of the animals in each paradigm were infused with saline and half with scopolamine. The US was a 1-s, 0.8-mA footshock, and the CS was a continuous 70-dB, 22-kHz tone.

The CS durations were identical to those used in a similar study that examined the effect of scopolamine infusion into PR on trace fear conditioning [30]. For trace conditioning, a 10-s CS was separated from the US by a 16-s trace interval. In long-delay conditioning, a 27-s CS co-terminated with the US. For both trace and long-delay, the interstimulus interval (ISI) between the CS onset and US onset was 26 s. For short-delay conditioning, a 15.6-s CS co-terminated with the US (ISI = 14.6 s). All three procedures consisted of 10 trials, separated by a mean intertrial interval (± SD) of 290±17 s.

The conditioning procedure was delivered using automated computer software (TestPoint; Measurement Computing). The CS was played using an RP2.1 real-time processor connected to an ES1 electrostatic speaker and an ED1 electrostatic loudspeaker driver (Tucker-Davis Technologies). A heterodyne bat detector (Mini-3; Noldus Technology) transformed the CS into an audible frequency that allowed the experimenter to monitor cue presentations.

### Conditioning and Testing Apparatus

Conditioning and testing took place inside two rectangular Coulbourn chambers (29 cm×25.5 cm×32 cm). Chambers were enclosed by sound-attenuating containers, which were located in separate rooms. Conditioning and context testing were performed in Chamber A, which was brightly lit, scented with acetic acid, and contained a stainless steel grid floor. A Coulbourn precision animal shocker delivered the footshock through the grid floor. Cue testing was performed in Chamber B, which was completely dark and was scented with Febreeze. The flooring was made of linoleum and the walls were covered in a laminated floral design. An infrared camera (Weldex) in each chamber permitted the monitoring and recording of behavior.

### Behavioral Procedures

Fifteen to 20 min after infusions, each animal was placed in Chamber A. Following a 2-min acclimatization period, the trace, long-delay, or short-delay conditioning procedure was initiated. Animals remained in the chamber for 60 s after the final shock before returning to their home cage. On each of the following 2 days, animals underwent one of two tests. Cue and context tests were done in counterbalanced order. For the cue test, animals were placed in Chamber B for 12 min, which included an initial 2-min pre-CS period, 6 min of continuous CS presentation, and a 4-min post-CS period. This cue-presentation procedure, which is commonly used in trace fear conditioning experiments [15], [30], [38]–[41], provides sufficient data for analyzing both delay and trace conditioning. For the context test, animals were placed in Chamber A for 8 min. All testing was recorded for later analysis of freezing. Freezing behavior, which was measured using custom video analysis software [15], included any period of immobilization lasting at least 3 s.

### Histology

After completion of testing, animals were deeply anesthetized with isoflurane vapors (30% v/v, Butler Schein) in 1,2-propanediol (Sigma-Aldrich). A small marking lesion was made bilaterally through the guide cannulae by passing current between a ground wire and an insulated wire inserted into the guide cannula (5 s, 100 µA; Lesion Maker, Grass Instruments). Animals were perfused transcardially with 100 ml of 0.01 M PBS followed by 100 ml of 4% paraformaldehyde. Brains were removed and placed in paraformaldehyde overnight and then transferred to a 30% sucrose solution for cryoprotection. After at least 3 d, brains were sliced coronally (70 µm) using a freezing microtome (American Optical). Sections were mounted and Nissl stained using cresyl violet (Kodak) to verify cannula placement.

### Data Analysis

Freezing behavior during each conditioning and testing session was analyzed in 1-min time bins. For the context test, analysis was performed on the mean percent freezing during all 8 min of the test. For the cue test, analysis was done on the mean percent freezing during the 6-min cue presentation. The results were analyzed with 2-way analyses of variance (ANOVAs), followed by planned comparisons using two-tailed *t* tests. Effect sizes were measured using Cohen’s *d,* calculated by dividing the difference between the means by the pooled standard deviation [42].

## Results

### Verification of Cannula Placements

**Figure 2:**
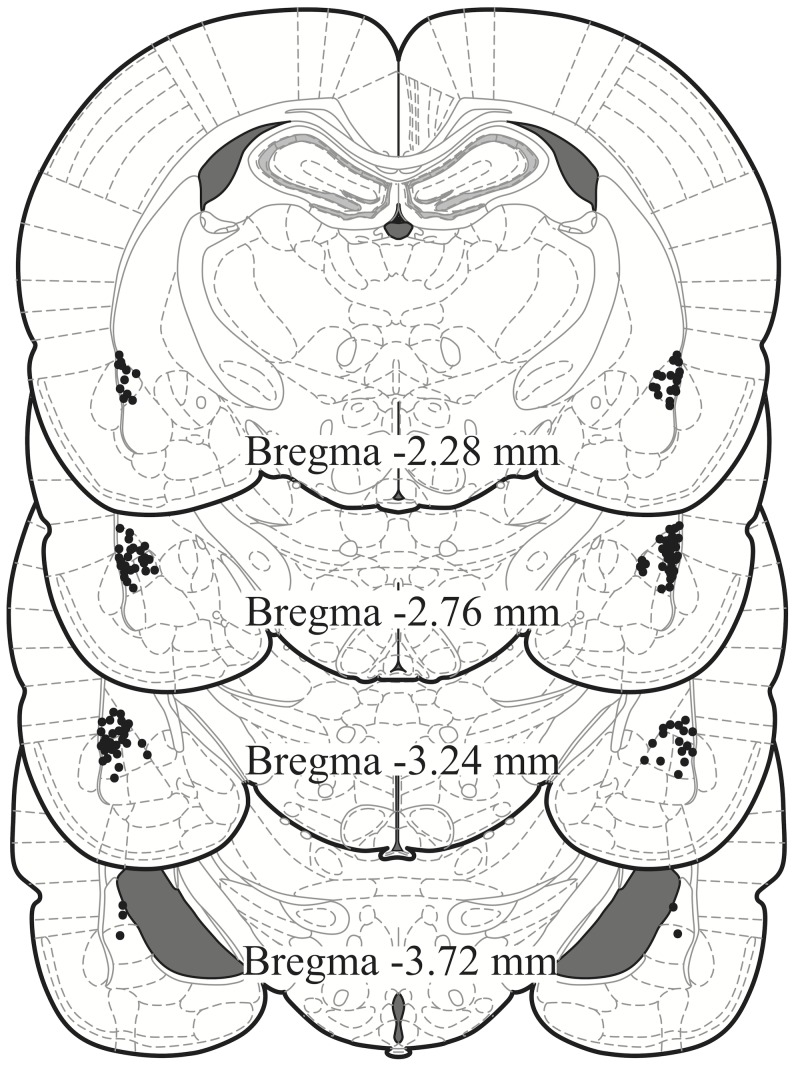
Localization of cannulae. Dots represent the tips of the infusion cannulae in the 69 rats that were included in the data analysis. Each rat is represented by two dots, one in each hemisphere. Plate numbers designate the distance posterior to bregma [37].

Cannula placements were assessed for all 90 rats. Only animals that had both cannulae in LA were included in the analysis (Fig. 2). Twenty-one rats were excluded, leaving a total of 69 rats for data analysis. The numbers of rats in each condition were as follows: scopolamine trace, *n = *15; scopolamine long-delay, *n = *11; scopolamine short-delay, *n = *8*;* saline trace, *n = *11; saline long-delay, *n = *12; saline short-delay, *n = *12.

### Baseline Freezing Levels

To exclude the possibility that the infusion agent or the conditioning paradigm affected baseline freezing levels, ANOVAs were performed on baseline freezing before the CS presentation both in conditioning and in the cue test. There was no significant main effect of infusion agent and no significant interaction between the agent and the paradigm, *p*s >.05, indicating that neither variable was associated with altered baseline freezing levels.

### Scopolamine Effect on Cue Conditioning

**Figure 3:**
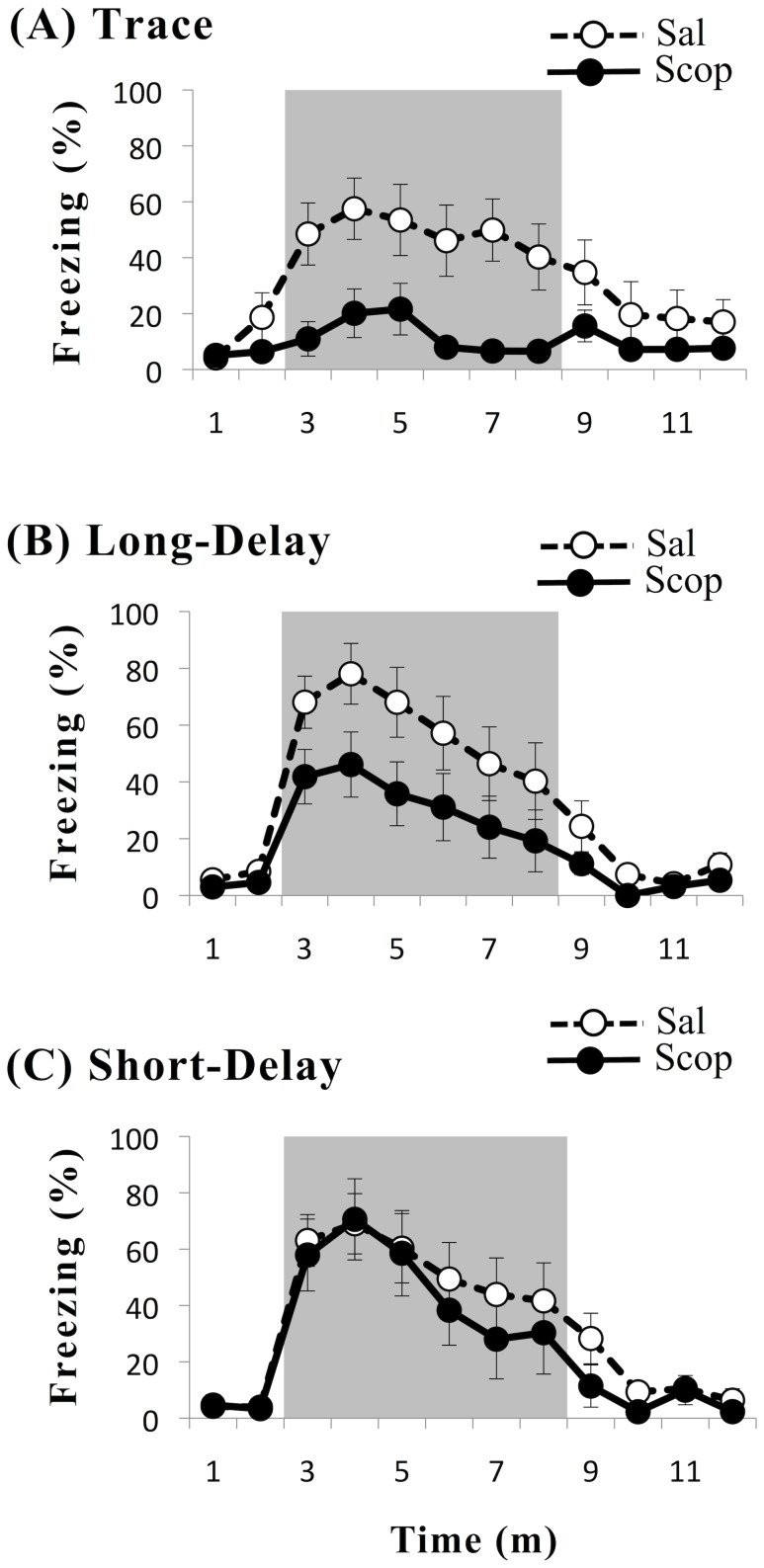
Results of cue test. The time series shows the percentage of time spent freezing (± SE) during the 12-min cue test. Open circles are from the control group (Sal) that received a saline infusion. Closed circles are from the experimental group (Scop) that received a scopolamine infusion. Parts A, B, and C, respectively, are plots from the trace, long-delay, and short-delay conditioned animals. Gray shading represents the 6-min period of continuous CS presentation. The only statistically significant difference was between the saline-infused (open circles) and scopolamine-infused (closed circles) animals in the trace conditioning paradigm. Thus, scopolamine infusion blocked trace but not delay fear conditioning.

Figure 3 graphs the time-course of freezing during the cue test for scopolamine-infused and saline-infused rats in each of the three conditioning paradigms. Overall, the mean percent freezing (± SE) was significantly lower in scopolamine-infused rats (30.9±5.6) than in saline-infused control animals (54.5±5.4), *F*(1,63) = 9.26, *p* = .003. There was no significant difference in freezing among the conditioning paradigms, *F*(2,63) = 2.56, *p* = .085. Likewise, there was also no significant interaction between the conditioning paradigm and the infusion agent, *F*(2, 63) = 1.21, *p* = .304.

Planned comparisons were performed to contrast the saline-infused and scopolamine-infused subjects in each conditioning paradigm. In the trace-conditioning group (Fig. 3A), the mean percent freezing was significantly lower among animals that received scopolamine infusions (12.30±6.56) than in control animals that received saline infusions (49.28±7.66), *t*(24) = 3.66, *p* = .001. The scopolamine infusion almost completely blocked trace conditioning (Fig. 3A, solid circles). The drug effect size was large [42], *d = *1.4.

In the long-delay conditioning group (Fig. 3B), the mean percent freezing did not differ significantly between scopolamine-infused (33.03±10.53) and saline-infused (59.68±10.09) animals, *t*(21) = 1.83, *p* = .082. A post hoc *t*-test of freezing during the first two minutes after the CS onset was also not significant, *p*>.05. The infusion clearly did not block long-delay conditioning (Fig. 3B, solid circles). Similarly, in the short-delay conditioning group (Fig. 3C), there was no significant difference in freezing between scopolamine-infused (47.29±12.41) and saline-infused (54.56±10.13) animals, *t*(18) = 0.45, *p = *.655. The time course of freezing was virtually identical in the two infusion groups during the first few minutes after the CS onset (Fig. 3C). In summary, scopolamine significantly decreased freezing to an auditory cue in trace but not long-delay or short-delay conditioning.

**Figure 4:**
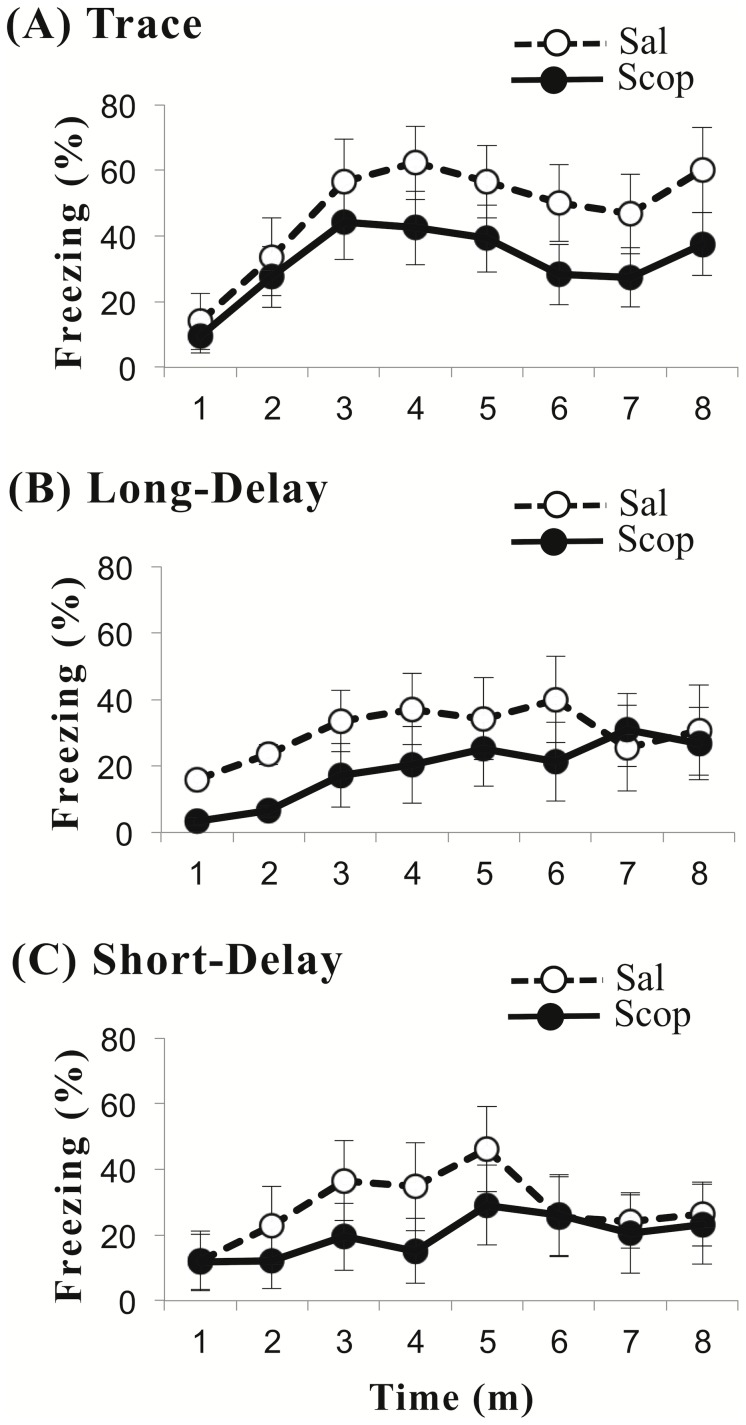
Results of context test. The time series show freezing, expressed as a percentage of total time (± SE), during the 8-min context test. Open circles are from the control group (Sal) that received a saline infusion. Closed circles are from the experimental group (Scop) that received a scopolamine infusion. Parts A, B, and C, respectively, are plots from the trace, long-delay, and short-delay conditioned animals. There were no statistically-significant differences between saline-infused and scopolamine-infused animals.

### Scopolamine Effect on Context Conditioning

Figure 4 plots the time-course of freezing during the context test for scopolamine-infused and saline-infused animals in each conditioning paradigm. ANOVA revealed no significant main effect of paradigm, *F*(2,63) = 2.80, *p* = .069, or infusion agent, *F*(1,63) = 3.44, *p* = .068, and no significant interaction between the paradigm and agent, *F*(1,63) = .094, *p* = .911. Planned comparisons also revealed no significant differences between scopolamine-infused and saline-infused animals in trace conditioning, *t*(24) = 1.34, *p* = .192 (Fig. 4A), long-delay conditioning, *t*(21) = 1.06, *p = *.301 (Fig. 4B), or short-delay conditioning, *t*(18) = .860, *p = *.401 (Fig. 4C). Thus, scopolamine infusion had no significant effect on context conditioning.

## Discussion

### Brief Summary

The present study provides clear evidence that mAChRs in the amygdala are required for trace fear conditioning. More specifically, pre-training scopolamine infusions into LA essentially blocked trace conditioning but had no significant effect on delay or context conditioning (Figs. 3 and 4). This pattern of results was uniquely anticipated by the EPF hypothesis for trace fear conditioning. [16], [17], [25], [30].

### Localization of Scopolamine Effect

All infusion sites were localized within LA (Fig. 2). Based on a diffusion radius ≥1 mm [43], [44], we can be confident that LA was thoroughly infused. Presumably, other subnuclei of the amygdala were also affected. However, thus far, EPF in the amygdala has only been reported in LA. The possibility of drug spread from LA to PR is of interest because EPF has been discovered in layer II/III of PR [17] and because scopolamine infusion into PR is known to impair trace fear conditioning without affecting delay or context conditioning [30].

Several considerations suggest that scopolamine diffusion from LA to PR might not be a critical issue. First, the two regions are separated by the external capsule, which can limit diffusion [44]. Second, EPF was discovered in superficial layers of PR, which are furthest from the amygdala. Third, there are reciprocal connections between PR and LA along the full length of PR [45], [46], which extends ∼5 mm beyond the posterior edge of LA. Thus, even if the portion of PR that lies laterally adjacent to LA were affected, this would not prevent EPF in more posterior regions, which also communicate with LA. Finally, as described below, trace fear conditioning has been proposed to be supported by EPF in a spatially-distributed system that includes both LA and PR, as well as other structures [17], [25].

### Short-Delay, Long-Delay, and Trace Conditioning

Most studies of the amygdala and fear conditioning have examined what is here termed "short-delay conditioning," more commonly referred to simply as “delay conditioning.” Therefore, the key group comparison in the present study was between trace and short-delay conditioning (Fig. 3A,C). Long-delay conditioning was added as a control for possible ISI-specific versus trace-specific effects [38], [47]. Although scopolamine infusion produced no significant effect in the long-delay conditioning group, the time series shown in Figure 3B invites further consideration of this paradigm. Unfortunately, the interpretation of drug effects in this group is limited by the fact that little is known about long-delay fear conditioning and even less is known about its relationship to trace fear conditioning.

Whereas the CS and US overlap temporally in both short- and long-delay conditioning paradigms, in some respects long-delay conditioning is more similar to trace conditioning than to short-delay conditioning. Long-delay and trace conditioning both share a similar developmental trajectory [48], [49], they both depend upon hippocampal function (when an extremely long delay is used) [50], and they both promote adult neurogenesis [51]. Conceivably, EPF could help support long-delay fear conditioning. Suppose, for example, that the prolonged tone used in long-delay conditioning resulted in spike-frequency accommodation in the auditory pathways to LA. EPF might help counteract this stimulus habituation.

### Comparisons with Previous Studies

A recent study reported that pre-training infusions of the mouse amygdala with a GABA_A_ receptor agonist (muscimol) produced deficits in delay conditioning and context conditioning, but had no effect on trace fear conditioning [52]. Based on our assumed diffusion radius (≥1 mm) and the size of the mouse brain, the infused muscimol should have reached all parts of the amygdala as well as adjacent cortices. The conclusion from this study was that the insertion of a temporal gap between the CS and US can generate amygdala-independent fear conditioning. An alternative circuit, which bypasses the amygdala, was proposed to entail projections from ventral HC to prelimbic cortex, and from there to the periaqueductal grey (PAG) region. The ventral PAG is known to control freezing behavior [53].

The present results (Fig. 3) obviously offer no support for this interesting hypothesis. Furthermore, we have no explanation for the apparently-contradictory findings. To place this discrepancy in a larger context, we note that several other pre-training manipulations of amygdala function also impair trace conditioning in rats. These include muscimol inactivation [31], [32], 6-hydroxydopamine lesions [33], and ibotenic acid lesions [33]. In addition, post-training infusions of the amygdala with anisomycin, a protein synthesis inhibitor, severely impair retention of both trace and delay fear conditioning [54].

In addition to reinforcing these previous results regarding the role of the rat amygdala in trace fear conditioning, the present study demonstrates that scopolamine infusion into the amygdala can block trace conditioning without significantly affecting delay and context conditioning. As indicated earlier, scopolamine infusion into PR produces the same pattern of results [30]. Notably, PR lesions impair both trace and context conditioning [15], [25]. Similarly, infusions of mAChR-antagonists into EC and HC impair trace but not delay conditioning [16], [36], whereas lesions of these same areas attenuate both trace and context conditioning [16], [34], [55].

In contrast to these selective effects of scopolamine infusions, other manipulations of rat amygdala function impair both delay and context conditioning. For example, amygdala lesions universally attenuate both delay and context fear conditioning [56]–[58]. In addition, both delay and context conditioning are markedly impaired by intra-amygdalar infusions of a number of pharmacological agents. In rats, these have included muscimol [59], an NMDA-receptor antagonist [60]–[62], inhibitors of plasticity-related signaling molecules [63]–[65], and antisense oligodeoxynucleotides that interfere with gene expression [66]–[68].

### Muscarinic Theory of Trace Fear Conditioning

LA is now the fourth structure in which mAChR-antagonism has been shown to impair trace but not short-delay fear conditioning, the other three being EC [16], PR [30], and HC [36]. All four brain regions also contain mAChR-dependent, persistent-firing neurons [11], [14], [17]–[21], [23], [24], [26], [27]. Importantly, reciprocal connections among these four structures [45], [46], [69], [70] should enable them to cooperate in support of a local transient memory system [17], [19], [25]. The fact that blocking mAChRs in any one of these structures interferes with trace conditioning suggests that the entire system must be concurrently operational. One possible function of EPF in this system could be to support reverberating activity in recurrent neural circuits [25]. As noted earlier, the concept of Hebbian reverberations [10] has been the standard model for transient trace memory. EPF could help drive these reverberations or it might function independently of them.

Other brain regions may additionally be involved. Persistent-firing neurons have been observed in the anterior cingulate cortex (ACC) [22] and ACC lesions similarly impair trace but not delay fear conditioning [71]. Unknown still is whether mAChR blockade in ACC selectively impairs trace fear conditioning. Persistent-firing neurons have also been discovered in the postsubiculum, where they have been suggested to support the sustained activity of head-direction neurons in the absence of exteroceptive navigational cues [29].

The basal forebrain area furnishes the major endogenous source of ACh for PR, HC, EC, and LA [72]–[74]. Important and unresolved questions concern the neurophysiological mechanisms and psychological conditions that control firing in these cholinergic projection neurons. One possibility is that firing is regulated by projections from prefrontal cortex [75]–[77] in conjunction with stimulus novelty or selective attention. Substantial evidence indicates that cholinergic systems influence myriad cognitive processes, including cue detection, sustained attention, and memory encoding [75]. Acetylcholine levels in the brain fluctuate on multiple time scales ranging from seconds to minutes, which may enable concurrent modulation of a number of functions essential for transient memory [75]–[78].

The present study adds to the mounting body of evidence that mAChRs play a pivotal role in trace fear conditioning. Additional research is necessary to elucidate the precise role of ACh release in transient memory. The EPF hypothesis offers a rich source of novel research directions. Better tests of this hypothesis will become available as more is learned about the molecular biology and pharmacology of the ion channels that support EPF.
